# Explainable AI-prioritized plasma and fecal metabolites in inflammatory bowel disease and their dietary associations

**DOI:** 10.1016/j.isci.2024.110298

**Published:** 2024-06-17

**Authors:** Serena Onwuka, Laura Bravo-Merodio, Georgios V. Gkoutos, Animesh Acharjee

**Affiliations:** 1 Institute of Cancer and Genomic Sciences, University of Birmingham, Birmingham, UK; 2 Centre for Health Data Research, University of Birmingham, Birmingham, UK; 3 Institute of Translational Medicine, University of Birmingham, Birmingham, UK

**Keywords:** Bioinformatics, Endocrinology, Human genetics

## Abstract

Fecal metabolites effectively discriminate inflammatory bowel disease (IBD) and show differential associations with diet. Metabolomics and AI-based models, including explainable AI (XAI), play crucial roles in understanding IBD. Using datasets from the UK Biobank and the Human Microbiome Project Phase II IBD Multi’omics Database (HMP2 IBDMDB), this study uses multiple machine learning (ML) classifiers and Shapley additive explanations (SHAP)-based XAI to prioritize plasma and fecal metabolites and analyze their diet correlations. Key findings include the identification of discriminative metabolites like glycoprotein acetyl and albumin in plasma, as well as nicotinic acid metabolites andurobilin in feces. Fecal metabolites provided a more robust disease predictor model (AUC [95%]: 0.93 [0.87–0.99]) compared to plasma metabolites (AUC [95%]: 0.74 [0.69–0.79]), with stronger and more group-differential diet-metabolite associations in feces. The study validates known metabolite associations and highlights the impact of IBD on the interplay between gut microbial metabolites and diet.

## Highlights

• Fecal metabolites predict IBD more effectively than plasma (AUC: 0.93 vs. 0.74)
• Key features include plasma inflammatory markers, fecal nicotinic acid metabolites
• Diet-metabolite associations stronger in feces, and differ between IBD and non-IBD

## Graphical abstract

**Figure:**
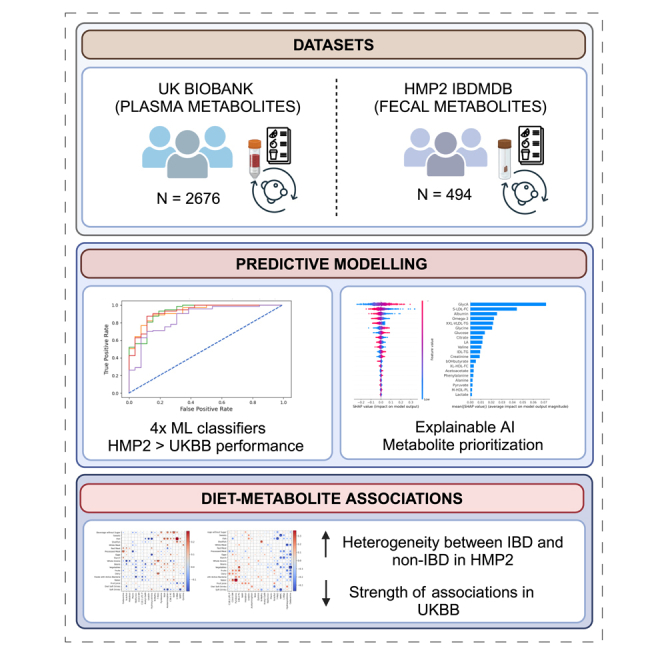

## Introduction

Inflammatory bowel disease (IBD), which primarily includes ulcerative colitis (UC) and Crohn’s disease (CD), is characterized by chronic gastrointestinal conditions that collectively affect approximately 5 million individuals as of 2019.^1^ Despite the rising global prevalence rates of IBD,^2^ its etiology remains elusive, with the main regulator of IBD pathogenesis believed to be the adaptive immune system as the main mediator of gut inflammation.^3^^,^^4^^,^^5^^,^^6^ This immune response is inherently linked to the genetic makeup of an individual; however, studies show varying proportions of heritability.^7^^,^^8^^,^^9^^,^^10^ Studies have also strongly linked IBD development with factors such as the gut microbiome,^11^ the use of antibiotics,^12^ and diet.^12^ Unraveling such biological complexity necessitates targeted-omics studies, with metabolomics recently helping to identify distinct disease-related patterns^13^^,^^14^^,^^15^^,^^16^^,^^17^ and key differences between individuals with IBD and those without.^18^^,^^19^^,^^20^ These differences have been observed as alterations in fecal short-chain fatty acids^21^ and serological lipids, such as cholesterol levels and its lipoprotein levels,^22^^,^^23^ and as changes in amino acid profiles, generally increased in feces^21^^,^^24^ and decreased in serum or plasma,^25^^,^^26^ as well as energy-related metabolites.^27^^,^^28^

As metabolomics is used to unravel these intricacies of IBD, it is increasingly being assessed in clinical practice, with AI-based modeling of metabolomics data integration helping identify potential metabolic markers that could be leveraged for therapeutic purposes. Promising studies have shown the power of machine learning (ML) in predicting IBD diagnosis, remission responses, and surgery risk,^29^^,^^30^^,^^31^^,^^32^ but in order for these approaches to be properly translated into clinical practice, issues regarding model interpretability and explainability need to be tackled. The better the performance of a model, the greater tendency for it to be increasingly complex, like in the case of ensembles and deep learning models, and not intrinsically interpretable, as in decision trees models. Therefore, balancing model performance with complexity is essential,^33^^,^^34^ with post-hoc explainability techniques,^35^ also known as explainable AI (XAI),^36^ emerging as key resources. The application of XAI in biomedical research particularly saw a surge in 2020, correlating to the rise of COVID-19 globally.^35^ However, the utilization of XAI in ML-based studies on IBD pathogenesis is an area that has received limited exploration and investigation.

Further, AI-identified important metabolites in IBD are also likely intertwined with diet, as the nutrients consumed by an individual play a crucial role in modulating numerous metabolic processes within the organism,^37^ and numerous studies have explored the relationship between diet and IBD. Diets rich in fiber have been linked to a lower risk of either the development of IBD or recurrence of symptoms after remission,^38^^,^^39^^,^^40^ while western-style diets characterized by high consumption of refined carbohydrates, red meat, high-fat foods, and ultra-processed foods have been found to elevate the risk of IBD.^41^^,^^42^^,^^43^ Additionally, dietary interventions for remission, including enteral nutrition for induction^44^^,^^45^^,^^46^ and specific carbohydrate diets^47^^,^^48^^,^^49^^,^^50^ for maintenance, have been explored as strategies for managing IBD. However, despite this wealth of research on diet associations with IBD, there exists a notable gap in understanding the interactions of diet directly with metabolic processes in IBD.

This study aims to fill these gaps by leveraging two publicly available datasets: the UK Biobank (UKBB)^51^ and the Human Microbiome Project Phase II IBD Multi’omics Database (HMP2)^52^ for metabolomics-based IBD prediction, applying XAI, and exploring the relationships between key metabolic profiles and dietary intake in IBD and non-IBD individuals. Results will potentially reveal important biomarkers for IBD, improve understanding of complex model predictions in IBD, and offer a nuanced understanding of the intricate relationship between metabolism, diet, and IBD pathogenesis. Ultimately, these findings may pave the way for the development of more targeted and effective interventions for individuals affected by IBD.

## Results

### Baseline data characteristics

**Figure 1:**
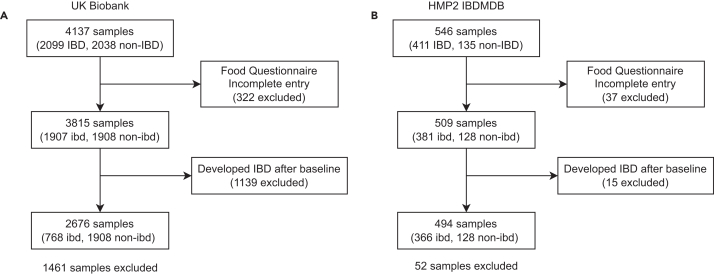
Sample exclusion flowchart The filtering process of samples of (A) the UKBB cohort and (B) the HMP2 cohort, based on their respective exclusion criteria.

A total of 1,461 and 52 samples were excluded for the UKBB and HMP2 cohort, respectively (Figure 1), with an average of 5.1 (SD = 1.4) samples per individual in the latter cohort. Baseline demographic characteristics of the resulting 2,676 samples of the UKBB, and 494 samples of the HMP2, are shown in Table 1. About one-third of the UKBB samples (768 samples) belonged to the IBD class, while an equivalent proportion of the HMP2 cohort consisted of non-IBD samples (128 samples). The median ages were 59 and 21 for the UKBB and HMP2 cohorts, respectively. They both had a gender distribution split of approximately 50% (52.1% females in the UKBB plasma dataset; 49.0% females in the HMP2 feces dataset), with over 90% belonging to the white race in both cohorts, and within each class. In the HMP2 cohort, more than half of the IBD samples consisted primarily of individuals who had received a diagnosis within the past year. Conversely, an equivalent proportion of the UKBB IBD samples consisted of individuals who had been diagnosed at least 6.7 years ago.

**Table 1:** Baseline demographic characteristics

	UKBB^a^ Cohort Characteristics				HMP2^b^ Cohort Characteristics			
	Total	Non-IBD	IBD	*p* value	Total	Non-IBD	IBD	*p* value
Participants	2676	1908	768		494	128	366	
Median age (years)	59.0 (52.0–64.0)	59.0 (52.0–64.0)	60.0 (51.0–64.0)	0.42	23.0 (14.0–43.0)	23.0 (13.0–55.0)	22.0 (15.0–41.0)	0.83
Female participants (%)	1394 (52.1)	985 (51.6)	409 (53.3)	0.47	242 (49.0)	56 (43.8)	186 (50.8)	0.20
White^c^ race (%)	2569 (96.0)	1827 (95.8)	742 (96.6)	0.36	445 (90.1)	123 (96.1)	322 (88.0)	0.01
Median years since diagnosis (IQR, years)			6.7 (3.0–11.3)				0.0 (0.0–12.0)	

a UKBB: UK Biobank.

b HMP2: Human Microbiome Project Phase II.

c For UKBB, this includes “British,” “any other white ethnicity,” and “Irish;” For the HMP2, this includes only “White.”

### Model performance

**Figure 2:**
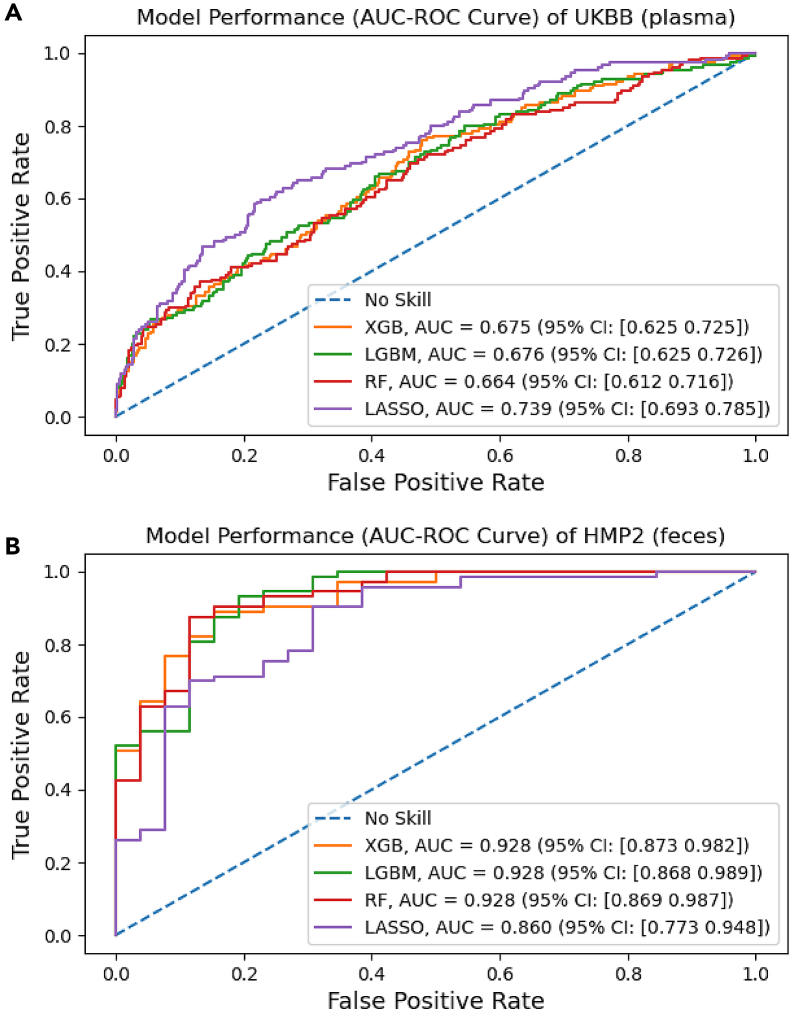
Model performance comparison The AUC_test_-ROC curves of the optimized classifiers for (A) the UKBB data and (B) the HMP2 data, with the blue dashed line representing a model performance that had no skill at all for comparison, are illustrated here (ROC, receiver operating characteristic; XGB, extreme gradient boosting; LGBM, light gradient boosting machine; RF, random forest; LASSO, least absolute shrinkage and selection operator). Data are represented as “mean (confidence interval).” See also Figure S1.

The UKBB metabolome data of 37 features were used to train the four classifiers used in this study: extreme gradient boosting (XGBoost), light gradient boosting machine (LGBM), random forest (RF), and least absolute shrinkage and selection operator (LASSO) regularization. The plasma metabolites of the UKBB cohort did not generally yield good model performances. LASSO emerged the top among the four classifiers, achieving an area under the curve (AUC) test score of 0.74 (95% CI: 0.69–0.79), while the other ML methods scored about 0.67 (Figure 2). With an optimal grid alpha value of 0.001, LASSO performed excellently in identifying individuals without IBD, having a specificity of 0.97 (Table 2). However, it was not effective in capturing all true IBD cases, as only about 14% of the IBD samples were correctly predicted (recall = 0.143). However, when it did predict IBD, there was an approximate 69% likelihood that it was truly IBD (precision = 0.688).

**Table 2:** Performance metric scores of the UKBB and the HMP2

Dataset (matrix)	Classifier	Test AUC (%)	Specificity (%)	Recall (%)	Precision (%)	F1 (%)
UKBB (plasma)	XGB	0.675	0.969	0.175	0.692	0.280
	LGBM	0.676	0.963	0.227	0.714	0.345
	RF	0.664	0.974	0.188	0.744	0.301
	LASSO	0.739	0.974	0.143	0.688	0.237
HMP2 (feces)	XGB	0.928	0.654	0.959	0.886	0.921
	LGBM	0.928	0.654	0.986	0.889	0.935
	RF	0.928	0.615	0.973	0.877	0.922
	LASSO	0.858	0.692	0.890	0.890	0.890

Among the HMP2 data, all the classifiers trained on the 160 fecal metabolites performed well. However, LASSO notably performed the least (AUC = 0.86), with all ensemble methods achieving about the same AUC score of 0.93 (Figure 2). Considering the performance of the ensembles across the other metrics, LGBM emerged as the preferred classifier, yielding an AUC test score of 0.93 (95% CI: 0.87–0.99) with the following optimal hyperparameters: 'colsample_bytree' = 0.8, 'learning_rate' = 0.1, 'max_depth' = 20, 'num_leaves' = 31, and 'subsample' = 0.8. This indicates that the model was effective at distinguishing between IBD and non-IBD cases, with an F1 score of 0.94 reflecting a balance between precision and recall (Table 2). Out of all positive predictions, up to 89% were truly IBD, with the model exceptional in capturing true IBD cases with a 99% recall rate. Nevertheless, the model’s specificity score of 0.65 suggests that its ability to accurately identify non-IBD cases was limited. Essentially, the model had more errors in correctly identifying non-IBD cases than in missing IBD cases.

### Feature selection and interpretation

**Figure 3:**
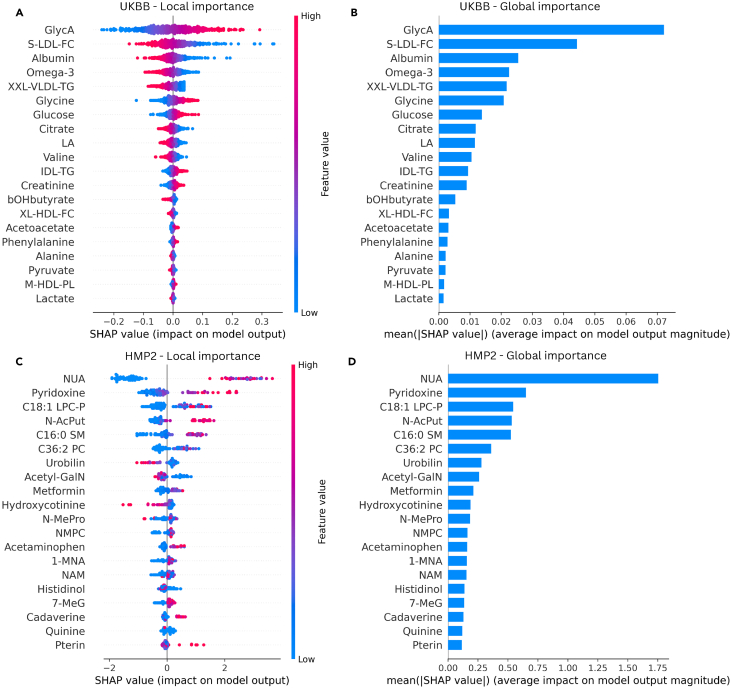
SHAP-based feature selection and model interpretation This figure showcases ranked SHAP summary plots of model predictions. (A) and (B) depicts LASSO-based plots of the UKBB cohort, and (C) and (D) depicts LGBM-based plots of the HMP2 cohort. The features shown are the top 20 metabolites of each model based on SHAP values. In the local importance summary plot for SHAP values (left), the samples are represented as the colored dots, with the color determined by the value it has for that feature. A positive SHAP value corresponds to a positive impact on the model, driving the algorithm toward prediction of the positive class, and vice versa. In the global importance summary plot for mean absolute SHAP values (right), features higher in rank correspond to a greater number of samples with SHAP values significantly deviating from zero, either positively or negatively (LASSO, least absolute shrinkage and selection operator; LGBM, light gradient boosting machine). Note: (A) and (B) illustrate summary plots for prediction non-specific to class, as linear models typically output a single set of SHAP values, while (C) and (D) represent a tree model, which produce class-specific SHAP values and thus, are specific to the positive class, IBD. See also Figures S1 and S2.

Following model predictions, Shapley additive explanations (SHAP) was applied to interpret such predictions, focusing on the contributions of the top 20 features at local and global levels. In the UKBB model, the top 20 out of the 37 metabolites contributed 99.6% of the cumulative absolute mean SHAP values, resulting in a top-to-bottom ratio of 44.51. SHAP local impact and global importance plots were generated for these features (Figure 3). Overall, these top metabolites included markers of inflammation (GlycA), lipoprotein subclasses (S-LDL-FC, XL-HDL-FC, and XXL-VLDL-TG), fatty acids (omega-3 and LA), amino acids (glycine and valine), energy metabolism (glucose and acetone), and waste product creatinine. Notably, GlycA emerged as the most discriminative metabolite with a substantial lead over the next two closely ranked features, S-LDL-FC and albumin, as observed in the global importance plot (Figure 3B). These results are complimented by the LASSO analysis in R that show GlycA, albumin, S-LDL, as well as omega-3 and glycine, to be the top features of the 400 bootstrapped models (Figure S1). Analyzing the local model impact plot (Figure 3A), higher values of GlycA, glycine, glucose, and creatinine were observed to drive the model toward prediction of the positive class, IBD. Contrarily, IBD prediction was driven by lower values of S-LDL-FC, omega-3, and albumin.

In the HMP2 model, the top 20 metabolites out of the total 160 contributed 60% of the cumulative absolute mean SHAP values, with a top-to-bottom ratio of 81.09. Among many other classes, these metabolites included vitamin B3 compounds (nicotinuric acid [NUA], N1-methyl-2-pyridone-5-carboxamide [NMPC], 1-methylnicotinamide [1-MNA], and nicotinamide [NAM]), and lipids (a lysophosphatidylcholine [C18:1 LPC-P], a sphingomyelin [C16:0 SM], and a phosphatidylcholine [C36:2 PC]). Remarkably, NUA emerged as the most discriminatory by an extreme margin (Figure 3D), with elevated levels driving IBD prediction (Figure 3C). Interestingly, no non-IBD sample contributed to IBD prediction, while a mix of non-IBD and IBD samples having low NUA values contributed to non-IBD prediction (Figure S2). Similar to NUA, most other top metabolites like pyridoxine, and n-acetylputrescine (N-AcPut) had higher values associated with IBD. In contrast, elevated levels of features like urobilin and hydroxycotinine drove prediction of the negative class, non-IBD. Complementary to these results, six of the top 20 SHAP-ranked metabolites, NUA, pyridoxine, N-AcPut, urobilin, C16:0 SM, and hydroxycotinine, were among the features of the 400 bootstrapped LASSO models in R that appeared the most, based on a threshold (Figure S1).

### Diet-metabolite correlations

**Figure 4:**
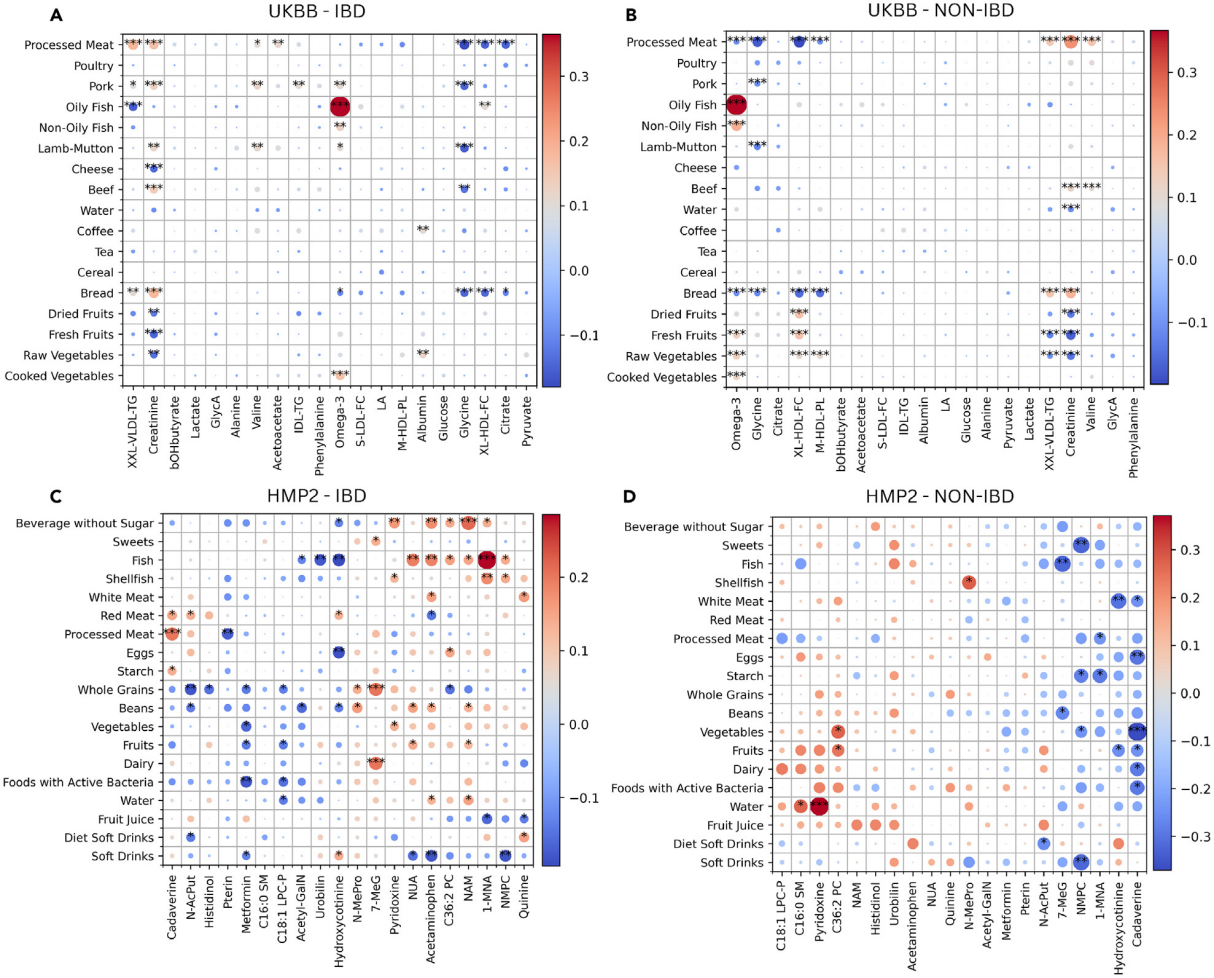
Diet-metabolite associations This figure shows diet-metabolite heatmaps of the SHAP-calculated top 20 metabolites for the (A) IBD class of the UKBB, (B) non-IBD class of the UKBB, (C) IBD class of the HMP2, and (D) non-IBD class of the HMP2. Circles are color-coded to represent Spearman correlation values, with the circle size indicating the strength of correlation. Significance levels are denoted by asterisks (∗∗∗: FDR < 0.001, ∗∗ 0.001 ≤ FDR < 0.01, ∗: 0.01 ≤ FDR < 0.05). The metabolites on the x-axis are “ward” clustered. See also Figures S4 and S5.

After the top SHAP-ranked features were correlated with their corresponding dietary components for each case-control group, the diet-metabolite correlations were found to be similar across both groups in the UKBB cohort. As depicted in Figures 4A and 4B, individuals with IBD had 36 correlations, while those without had 32, all being significant. Moreover, 90% of the correlations fell within the range of (−0.091, 0.109) for the IBD group, and (−0.094, 0.094) for non-IBD group. Dietary wise, omega-3 fatty acid had the strongest correlation across both groups, displaying significantly moderate correlation levels with oily fish, with levels up to three times higher than average in each group (in IBD: r = 0.365, and in non-IBD: r = 0.367; false discovery rates [FDR] < 0.001; refer to Table S4 for all correlation and FDR values). Similarly, across both groups, glycine was significantly negatively correlated with red meat intake (processed, pork, lamb, and beef), although weak. However, there was a considerable difference; certain food-metabolite correlation patterns observed were stronger among those without disease. Particularly, among non-IBD individuals, omega-3 and XL-HDL-FC exhibited a pattern opposite to that of creatinine and XXL-VLDL-TG across processed meat, bread, fruit and vegetable intake. For instance, XL-HDL-FC in the non-IBD group was negatively correlated with bread (r = −0.156, FDR < 0.001) while creatinine showed a positive correlation (r = 0.182, FDR < 0.001). However, among individuals with IBD, these correlations were notably diminished, with only creatinine showing significant associations across the aforementioned food groups.

In contrast to the minor variations observed across both IBD and non-IBD groups in plasma metabolite and diet interactions, the differences in fecal-diet interactions in the HMP2 cohort were much more pronounced. In this cohort, the non-IBD group had more correlations (R = 159) compared to the IBD group (R = 111). Individuals without IBD exhibited correlations stronger than those with IBD (Figures 4C and 4D), although only 22 out of the 156 correlations were significant, while in just over half of the 111 IBD correlations, 65 were significant. However, this is likely due to IBD samples being about three times the amount of non-IBD samples. Nonetheless, most correlations within this cohort were stronger than the correlations observed in the UKBB cohort, with 90% of the HMP2 correlations falling within the range of (−0.139, 0.149) for the IBD group, and (−0.224, 0.191) for the non-IBD group. Another notable difference was metabolites expressing opposing or non-existent associations for the same food group between IBD and non-IBD. For instance, while fish was significantly positively correlated with 1-MNA in the IBD group (r = −0.285, FDR < 0.001), it showed non-significant negative association in the non-IBD group (r = 0.192; refer to Table S5 for all correlation and FDR values). Further, water with pyridoxine (r = 0.371, FDR < 0.001) and cadaverine with vegetables (r = −0.371, FDR < 0.001), which tied for strongest correlation among the non-IBD samples, exhibited either weak or invalid correlation values among the IBD samples (both r = 0.065). Beyond these overview distinctions, there were a few diet-metabolite associations that remained similar across both groups, albeit with varying strengths. For instance, NMPC was significantly inversely correlated with soft drink consumption in both groups (IBD: r = −0.183 and non-IBD: r = −0.323, 0.001 < FDR ≤ 0.01), and hydroxycotinine demonstrated negative correlations with beans intake (IBD: r = −0.136, 0.01 ≤ FDR < 0.05 and non-IBD: r = −0.188). Further, some metabolites clustered in both groups, like lipid-related metabolites, C18:1 LPC-P and C16:1 SM and some vitamin B3 metabolites, NMPC and 1-MNA, indicating that they undergo similar metabolic patterns in association with diet, regardless of how different the associations may be with disease or without.

## Discussion

### Model performance

In the UKBB data, LASSO outperformed the other ML methods, which were all ensembles. This is likely due to a number of possibly redundant features included in the ensemble models, which were assigned zero weights in the LASSO model, which automatically filters out highly correlated variables. On the other hand, all the ensemble methods each outperformed LASSO in HMP2, highlighting the possible non-linear nature of the associations between outcome and features.

### Discriminatory plasma metabolites

SHAP XAI successfully explained the prediction of both cohort models and revealed the highly influential features of these models. The top features of the UKBB model included GlycA, albumin, sub-classes of high and low-density lipoprotein cholesterol forms, and omega-3 fatty acid. However, the model’s average AUC score (0.74) implies that these features are not very effective for classifying IBD within this cohort, which could indicate an insufficiency of these NMR-identified plasma metabolites to distinguish properly between IBD and healthy individuals, particularly in older adults. However, as the results are not as low as chance (0.50), it is reasonable to suggest that these highlighted key metabolites are involved in IBD pathogenesis, with findings validated by the existing literature in the following text.

For instance, a prior study also identified significantly elevated levels of GlycA, a well-established stable inflammatory marker,^53^^,^^54^^,^^55^ in individuals with active IBD compared to controls.^56^ Similarly, decreased levels of albumin, which has been stated to be an inverse biomarker for systemic inflammation,^57^ align with the UKBB model’s prediction of IBD. However, it is important to note that these two inflammatory markers are inclined to predict as IBD, only individuals with active IBD or individuals without IBD but are undergoing systemic inflammation at the time of testing.

Similar misclassifications may have occurred with other influential metabolites, such as LDL-C (S-LDL-FC), also known as “bad” cholesterol, where varying study findings suggest mixed LDL-C profiles in individuals with IBD, with some showing low levels,^58^^,^^59^ and others indicating high levels.^60^^,^^61^ However, the lower levels of HDL-C (XL-HDL-FC), or “good” cholesterol, observed in IBD predictions is common.^58^^,^^59^^,^^60^^,^^61^ Although for both lipoprotein cholesterols, the changes between the healthy and diseased were noticed more prominently with CD than with UC.^58^^,^^59^^,^^60^^,^^61^ Nonetheless, lipoprotein cholesterol levels being associated with IBD, and cholesterols having been associated with cardiovascular risk,^62^^,^^63^^,^^64^ add to the increasing number of findings linking IBD to cardiovascular disease risk.^65^^,^^66^^,^^67^ This suggests some shared lipid metabolisms underlying both disease types.

Further, in line with previous studies,^68^^,^^69^ lower levels of omega-3 fatty acid were found to be associated with IBD. However, these results are inconclusive concerning the role of omega-3 in IBD individuals. While it is known that omega-3 boosts one’s immunity,^70^ it has been potentially linked to a decreased risk of developing IBD^71^^,^^72^ and among IBD patients, has been linked to reduced intestinal inflammation^71^; it has also been speculated that the ratio of omega-3 to omega-6 is more crucial than the level of omega-3 itself.^68^ Further, it is unclear if depleted levels of omega-3 are causal, elevated levels provide protection, or supplementation is truly helpful for all individuals with IBD or only a subset of them.^71^^,^^73^

### Discriminatory fecal metabolites

While the plasma metabolites were not as effective in distinguishing between both healthy and diseased, the fecal metabolites in the HMP2 cohort showed much greater capabilities. Although achieving an AUC score of 0.93, higher than commonly published metabolomics-based predictions,^74^^,^^75^^,^^76^ may be inflated due to there being much more IBD than non-IBD samples, the high performance still underscores the potential importance of fecal metabolites in the pathogenesis or diagnostic assessment of IBD. This high discriminatory score prompts further investigation into the specific metabolites and underlying biological mechanisms driving this predictive power. Insights from the SHAP algorithm unveiled several metabolites that exhibited higher discriminatory abilities where variations in their levels—both high and low—were generally associated with class predictions. These key metabolites included metabolites of vitamin B3, phospholipid metabolites, and urobilin.

Vitamin B3, mainly represented as NA, with NAM as its main metabolite, is present in the body mainly through diet, and to a lesser extent, synthesized *de novo*. Fecal levels of NA have been found to be reduced in both CD and UC patients, compared to healthy controls, although more pronounced in CD patients.^77^ However, diminished levels of its metabolites (NUA, NMPC, 1-MNA, and NAM) were notably influential in predicting non-IBD cases. While low values contributed to IBD prediction, elevated levels were also involved, but increasingly so in the case of NUA.

The elevated NUA levels that strongly drove IBD prediction has been previously discovered to be due to a confounding drug effect.^78^ Only individuals with IBD displayed increased NUA levels (Figure S2). Further literature research then revealed that the NUA levels in the HMP2 cohort were confounded by intake of the 5-aminosalicylic acid (5-ASA) drug, the common first line of treatment for IBD patients,^79^^,^^80^ as only individuals that took it displayed increased NUA levels.^78^ Knowing this, the classifiers were re-run on the HMP2 data without considering NUA, and the ensemble models actually performed better on average, with the top classifier achieving an AUC score of 0.95 (Figure S5). This suggests that the potential bias introduced by NUA led to a slight decrease in model performance. However, the analysis without NUA was kept supplementary as model performance was not significantly impacted. Further, in this way, post-hoc measures are minimized, prioritizing realism and generalizability.

Nonetheless, higher levels of fecal NMPC and 1-MNA being associated with IBD prediction could be due to increased levels of NAM,^81^^,^^82^ a degradation product of nicotinamide adenine dinucleotide (NAD).^83^^,^^84^ NA and NAM metabolism, which involves NAD turnover that is increased in IBD,^85^ is currently a therapeutic target of manipulation for IBD patients,^86^ as multiple studies show the involvement of these metabolic processes and pathways with IBD.^81^^,^^87^^,^^88^^,^^89^

Phospholipid metabolites and urobilin were key discriminators as well. In line with existing studies,^75^^,^^90^ higher LPC (C18:1 LPC-P) was associated with IBD classification, with higher concentrations consistently shown to promote inflammation, injuring endothelial cells^91^ and damaging the epithelial barrier.^92^ Furthermore, elevated fecal levels of SM (C16:1 SM) were predictive of IBD, consistent with prior research showing increased SMs in IBD patients.^19^^,^^93^ Additionally, high urobilin levels were associated with non-IBD prediction, which is line with a previous study that found L-urobilin to be the most discriminative metabolite for the colitis phenotype in rats, with much higher concentrations being associated with the healthy, while non-existent in the colitis rats.^94^ Moreover, urobilin emerged the top discriminator when NUA was not considered (Figure S5). Nonetheless, with a recent study suggesting an elevated ratio of fecal sphingolipids to L-urobilin as an IBD-associated marker warranting further investigation,^74^ it may be the ratio to SMs and not the concentration in and of itself that is discriminatory.

### Reasons for the disparity between the plasma and fecal metabolites

There was notable discrepancy in performance between fecal metabolites (AUC = 0.93) and plasma metabolites (AUC = 0.74). Insights obtained from the LASSO modeling in R (Figures S1 and S5) also complement the ensemble results from python; the stronger association with IBD was found to be with the fecal metabolites of HMP2 as opposed to the plasma metabolites of the UKBB, evidenced by more features selected above the bootstrap threshold, as well as higher AUC values (mean: 0.896 ± 0.025 vs. 0.652 ± 0.017) in the 400 different bootstraps.

The difference between the predictive capabilities of the plasma and fecal metabolites can be attributed to a number of biological factors, associated with cohort differences. One could be age differences. Elderly individuals, of which the UKBB comprises, regardless of disease may exhibit similar metabolic patterns in the blood, particularly with cell membrane related lipids such as phospholipids,^95^ decreasing the discriminatory potential of the plasma metabolites. Moreover, the gut microbiome’s pivotal role in IBD,^96^ with its functions in inflammation regulation concerning the gastrointestinal tract^97^ and overall gut health, render fecal metabolites as the more potent markers for IBD diagnosis. To buttress this point, just recently, Raygoza et al. show that the composition of the gut microbiome is linked to the future development of IBD, particularly CD.^98^

### Diet-metabolome associations

The interplay between the metabolome and diet has been extensively studied,^99^ and also between diet and IBD as a whole,^100^ as mentioned in the introduction. However, there are gaps in understanding how the presence of IBD alters the metabolism of ingested food.

In the UKBB cohort’s plasma analysis, increased consumption of oily fish, notably rich in long-chain omega-3 polyunsaturated fatty acids, was associated with increased omega-3 levels in both groups. Moreover, the consumption of red meat has also been associated with reduced levels of glycine in the plasma.^101^^,^^102^^,^^103^ Studies also confirm the intake of fibers (fruit and vegetable), being positively correlated with omega-3 and HDL-C^104^^,^^105^^,^^106^ and negatively with creatinine^107^ observed among the non-IBD group. Considering that both groups had similar food intake distributions (Figure S3), and the overall metabolite profiles between the two groups did not generally exhibit visibly significant differences (Figure S4), a potential explanation for the slightly stronger correlation observed among those without IBD, could be the heterogeneity within the IBD cohort in terms of disease activity. A median time since diagnosis of 7 years, with the interquartile range going from 3 to 11 (Table 1), suggests varying disease activity levels. The subset of individuals with a longer disease history, potentially experiencing higher disease activity, could contribute to the observed metabolic profiles. During active IBD, or flare-ups in the case of inactivity, the body may respond to dietary intake in a distinct manner compared to those without active disease or without IBD. As this only possibly applies to a subset or multiple subsets within the whole IBD cohort, this variation could explain the weaker correlations observed within the IBD group.

In the HMP2 cohort however, the correlations were generally stronger than the ones observed in the UKBB, which is not surprising considering the well-established bidirectional relationship between the gut microbiome and diet.^108^^,^^109^^,^^110^^,^^111^ Further, there were only a few consistent diet-metabolite associations shared between the diseased and non-diseased groups of the HMP2 cohort among the top 20 metabolites, and the most prominent are currently not confirmed in literature (e.g., NMPC with soft drinks, and hydroxycotinine with beans). Further, differences across both groups was much more numerous among the fecal metabolites. This divergence extended beyond slightly diminished correlation strengths to near-zero or opposite correlation strengths. For instance, the relationship between fish intake and urobilin varied significantly, being positively associated among those with IBD but negatively among non-IBD samples, with similar strengths observed (0.216 vs. −0.177; refer to Table S5 for all correlation and FDR values). This underscores the notion that disease, in this case, IBD, exerts a biological influence on how individuals respond to diet. Just as explained with the plasma metabolites, the reduced strengths of correlation when compared to the healthy could also be due to differential responses to dietary intake due to different activity states of IBD. However, as IBD is also an immune-mediated disease, its presence may diminish diet-metabolite correlations more significantly than if it was absent in an individual, as the gut microbiome also regulates the immune system and affects systemic immune responses.^112^^,^^113^^,^^114^ Overall, considering the plasma and fecal dietary associations, the differential responses to diet when compared to the healthy, and even within IBD, possibly due to differing disease activity states, buttresses the need for more personalized approaches to dietary therapies for IBD management.

### Limitations of the study

This study is not without limitations. The inclusive diagnosis of individuals using ICD-9 and ICD-10 codes in the UKBB, without accounting for comorbidities, introduces potential confounding factors that may have impacted the model’s predictive accuracy, as these additional conditions might have strongly affected the metabolisms of some participants. The large UKBB sample size, while beneficial, may not have fully counteracted the effects of these confounding comorbidities. Another challenge arises from sample imbalance in both the UKBB and HMP2 cohorts, potentially introducing bias in model training and evaluation, particularly for the smaller HMP2 dataset, where the total number of samples was relatively small. This could be why the AUC score, which assesses a model’s ability to predict the positive class, IBD, was higher than expected (AUC = 0.93) considering that IBD is a multifactorial disease. Nonetheless, variations in the timelines of dietary information between the two datasets, an average of the past year for the UKBB compared to the past week for the HMP2 cohort, may have impacted the representativeness of subjects’ dietary habits during metabolic profiling, contributing to weaker correlations in plasma metabolites compared to fecal metabolites. Finally, the use of food questionnaires introduces a potential source of human error, as data accuracy relies on participants’ recall and honesty.

### Conclusion

Overall, our study addresses gaps in IBD research and lays a foundation for future studies by advancing our understanding of IBD pathogenesis through several key avenues. By leveraging the largely sampled UKBB data of over 2,500 individuals, to the best of our knowledge, this study represents the first-ever published comprehensive machine learning analysis of the plasma metabolome of IBD patients in the UKBB, offering unique insights. Further, by analyzing the well-documented fecal metabolites of the IBD samples of another major cohort, the HMP2, valuable contrasts are made available. Additionally, contributing to the work of developing diagnostic ML models drives us closer to developing an effective algorithm that predicts IBD before its onset, presenting a promising avenue for transforming patient care as more diverse risk factors such as smoking habits, and the presence of anti-saccharomyces cerevisiae antibodies in the blood are incorporated. Moreover, the use of XAI in the predictive models offers transparency, facilitating translation into clinical practice. Finally, the exploration of dietary associations illuminates the complex interplay between gut microbial metabolites and dietary factors in IBD, enhancing our understanding of disease mechanisms, and facilitating the development of targeted interventions.

## STAR★Methods

### Key resources table

**Table:** 

REAGENT or RESOURCE	SOURCE	IDENTIFIER
**Deposited data**		

Plasma metabolomics data	UK Biobank^51^	UK Biobank: 31224;
Fecal metabolomics data	HMP2 IBDMDB^52^	https://ibdmdb.org/results

**Software and algorithms**		

Codes generated for this study	This paper^142^	Zenodo: https://doi.org/10.5281/zenodo.11411432;
R	R Core Team^116^	https://cran.r-project.org/
tidyverse	Wickham et al.^139^	https://github.com/tidyverse/tidyverse
missForest	Stekhoven & Bühlmann^121^	https://github.com/stekhoven/missForest
caret	Kuhn^124^	https://github.com/topepo/caret
glmnet	Friedman, Hastie & Tibshirani^143^	https://github.com/cran/glmnet
Python	Van Rossum et al.^130^	https://www.python.org/downloads/
xgboost	Chen & Guestrin^126^	https://github.com/dmlc/xgboost
lightgbm	Ke, Meng, Finley, Wang, Chen, Ma, Ye & Liu T^127^	https://github.com/microsoft/LightGBM
sklearn.ensemble.RandomForestClassifier	Pedregosa et al.^131^	https://github.com/scikit-learn/scikit-learn
sklearn.linear_model.Lasso	Pedregosa et al.^131^	https://github.com/scikit-learn/scikit-learn
shap	Lunderg & Lee^134^	https://github.com/shap/shap
pingouin	Vallat^136^	https://github.com/raphaelvallat/pingouin
matplotlib	Hunter^137^	https://github.com/matplotlib/matplotlib
seaborn	Waskom^138^	https://github.com/mwaskom/seaborn

### Resource availability

#### Lead contact

Further information and requests for resources should be directed to and will be fulfilled by the lead contact, Dr Animesh Acharjee (a.acharjee@bham.ac.uk).

#### Materials availability

This study did not generate new unique reagents.

#### Data and code availability

• This paper analyzes existing, publicly available data. Accessibility details for these datasets are listed in the key resources table.
• This paper does not report original code. Derivative codes generated for this study have been deposited at Zenodo and is publicly available as of the date of publication. The DOI is listed as a citation in the key resources table.
• Additional information needed to reanalyze the data presented in this paper can be obtained from the lead contact upon request.

### Method details

#### Study design and participants

The blood-based metabolomics data in this study was retrieved from the UKBB. The UKBB is a large-scale cohort study conducted between 2006 and 2010, involving 500,000 consenting participants aged 40 to 69 from across the UK who provided detailed health information, and is approved by the North West Multi-centre Research Ethics Committee amongst others. At the data retrieval stage, 4,137 plasma samples (2,099 IBD and 2,038 healthy controls—no reported ICD-10 diagnosis—matched based on age and sex) were extracted from the UKBB. Matching was done using the “nearest” method which utilizes a greedy search to match each sample with their nearest neighbour. The distance was calculated using the Mahalanobis distance, which estimates the distribution closest for each point.^115^ This procedure was performed in R (v4.2)^116^ using the Matchlt package.^117^ IBD diagnosis was defined by corresponding International Disease codes (ICD-9: 555, 556; ICD-10: K50, K51) which included both Crohn’s disease (CD) and ulcerative colitis (UC). Self-reported cases of IBD (1461, 1462, 1463) were also considered.

The fecal-based metabolomics data in this study was gotten from the HMP2 IBDMDB. As part of the Integrative Human Microbiome Project, which is carried out under the National Institute of Health (NIH), the IBDMDB followed 132 consenting subjects over a year to generate comprehensive longitudinal molecular profiles of host and microbial activity during IBD. In this cohort, 546 fecal samples (411 IBD samples encompassing both CD and UC, and 135 control samples) were retrieved from the stools of 106 individuals, with an average of 5.6 samples per person (SD = 1.2), having been followed longitudinally for up to one year each.

The datasets of both cohorts were filtered to retain only individuals with complete data for their corresponding food questionnaire. Individuals who developed IBD after baseline were excluded in both cohorts, considering ‘Age at recruitment’ (Code: 21022) for the UKBB and ‘age at consent’ for the HMP2 as the baseline ages. These included IBD participants that had a history of disease of varying years (‘Median years since diagnosis’ in Table 1). Following filtering, multiple samples per participant within the HMP2 cohort of fecal samples were retained where applicable. This approach allowed for data to be maximally utilized while avoiding disproportionate emphasis on specific features, as temporal changes in the microbiome are more frequent and pronounced in IBD.^52^

#### Metabolite data pre-processing

Prior to any data processing, the UKBB dataset consisted of 168 metabolites including lipids and lipoproteins, fatty acids, and small molecules such as amino acids and metabolites related to fluid balance, inflammation, and glycolysis (refer to Table S1 for detailed information). The HMP2 dataset contained 176 metabolites which consisted of phospholipids, amino acids and derivatives, carnitines, and more (refer to Table S2 for detailed information). While the UKBB metabolomics data was generated using nuclear magnetic resonance (NMR),^118^ the HMP2 metabolomics data was generated using liquid chromatography mass spectrometry (LC-MS).^119^ Among the four LC-MS methods, metabolites derived from the HILIC-pos method were specifically selected. The data generation and quantification processes of the UKBB and HMP2 metabolomics data have been detailed elsewhere.^52^^,^^120^

All pre-processing steps were performed using R (v4.3.0).^117^ Imputation of the missing values, which accounted for less than 1% of the data, was employed on the UKBB datasets using “missForest” R package (v1.5).^121^ The 8.69% of the HMP2 data that were missing were imputed with half of the minimum positive value for each column according to common practices.^122^ As per common metabolomics procedures,^122^ pareto scaling was applied to both datasets to mitigate the influence of larger features while retaining cross-feature variance. Subsequently, a log2 transformation was conducted to address heteroscedasticity from the data and rectify skewed data distribution.^123^ To avoid errors in the log transformation for zero values, a pseudo-count of 1 was added to all values, since there were only a few zeros present in the UKBB dataset (< 1%) and none in the HMP2. Prior to removing the highly correlated features, features that represented sums of other cells and particle sizes were removed to enhance the efficiency of correlation analysis. The exclusion of sum-related features and particle sizes ensured that well-established features, such as sub-classes of HDL-C, were retained if possible, when found to be highly correlated with these metabolites. The highly correlated features of the remaining 156 features were then filtered out at a threshold of 0.9 based on the Spearman correlation method performed using the “caret” R package (v6.0.94).^124^ The resulting pre-processed datasets, derived from both UKBB (2676 samples x 37 features) and HMP2 (494 samples x 161 features) sources, were utilized for subsequent statistical analysis and machine learning tasks.

#### Diet data pre-processing

The UKBB dietary data encompassed a food frequency questionnaire (FFQ) about average diet intake in the past 12 months. The UKBB codes and corresponding names of these food groups are contained in Table S3. Two types of features were observed. The values of the numerical features (fruit, vegetables, coffee, tea, water, bread, and cereal intake) were used as is. Similar to a method applied by another study in handling the FFQ data in the UKBB,^125^ the frequency of the categorical features (processed and non-processed meat, and cheese intake) were assigned weights: never (0), less than once a week (0.07), once a week (0.14), two to four times a week (0.43), five to six times a week (0.79), once or more daily (1). This yielded 18 final diet features: 9 numerical and 9 weighted categorical. For the HMP2, the diet data consisted of a food questionnaire assessing food consumption frequency in the past week (Table S3). Weights were assigned as follows: no consumption (0), consumed {within the past [four to seven days (0.2), two to three days (0.56)], yesterday, one to two times (0.9), yesterday, three or more times (1)}. Refer to Figure S3 for the boxplots of food intake frequency distributions in each group for the UKBB and HMP2 cohorts.

#### ML classification of IBD and non-IBD

This study tested four machine learning methods, XGBoost,^126^ LGBM,^127^ RF,^128^ and LASSO^129^ in classifying disease and non-disease. XGBoost, LGBM, and RF make use of an ensemble of classification trees and combine the predictions from multiple individual decision trees to make more accurate and robust predictions, hence making them suitable for disease classification tasks. LASSO, on the other hand, is a popular regularization algorithm for logistic regression that helps reduce the feature space and highlight key associations.

All machine learning analysis carried out in python (v3.9.13)^130^ was done using the “scikit-learn” module (v1.0.2).^131^ The metabolomics datasets underwent an initial 80-20 train-test split. The optimization of training sets was performed using grid search (“GridSearchCV”) over predefined parameter grids of the various ML models to be tested. Stratified k-fold cross-validation with 5 folds was employed within the grid search ensuring robust model assessment and hyper-parameter tuning. The optimal grid model of each classifier was the model with the highest average validation AUC score. The best classifier of each dataset, based on the highest test AUC score metric, was passed into the SHAP explainer.

Using the same test and train split data produced from python, LASSO regularization^129^ was implemented with bootstrapping and out-of-bag sample assessment of the models^132^^,^^133^ in R to investigate its robustness and assess the stability of the top features. With the training set, 400 bootstraps were run with the ‘glmnet’ package in R, with LASSO (alpha = 1). Lambda was optimized using 10-fold cross-validation, with lambda chosen to be that which produces the highest AUC by 1 standard deviation. Evaluating the model at this lambda, the top features, which were chosen as those appearing more times than a threshold (average between the fourth and fifth quantile), and their coefficients were extracted and visualized (see Figures S1 and S5).

#### Running the explainable AI on the best classifier

Following training and testing, the best classifier and test set were introduced to the SHAP (SHapley Additive exPlanations) tool in Python (“shap” v0.41.0).^134^ Metabolites with the highest impact were identified using SHAP global importance plots. SHAP local impact plots that illustrate the contribution of each top metabolite to sample predictions were also generated. SHAP elucidates the contribution of each feature to the model's predictions, employing concepts from cooperative game theory to quantify feature importance.^135^ This approach not only enhances model interpretability but also provides insights into the decision-making process, thereby increasing transparency in the model's output.

#### Statistical analysis of the top metabolites

The top 20 metabolites based on SHAP-based ranking were then spearman-correlated with diet features using the “pingouin” Python package (v0.5.3).^136^ The results were visualized on a circle-style heatmap generated using “matplotlib” in Python (v3.5.2).^137^ Metabolites on the x-axis were clustered using the ‘ward’ method in the “seaborn” Python package (v0.11.2).^138^ Correlation values with absolute values above 0.1 were counted as valid. Only valid correlation values were considered significant, that is, their false discovery rates (FDR) being less than 0.05. Further, to visualize how well each top feature predicted the samples, faceted boxplot distributions of the SHAP values of IBD and non-IBD samples for the top metabolites of both cohorts were generated (Figure S2). Additionally, in order to also visualize the differences in metabolite profiles between the IBD and non-IBD classes, faceted boxplot distributions of both IBD and non-IBD groups of these top metabolites were generated using R packages, with differential metabolites calculated using the Wilcoxon rank sum test (Figure S4). All boxplots were created using the “ggplot2” R package (v3.4.2), located in the “tidyverse” library,^139^ and transformed into publishable-ready plots using “ggpubr” R package (v0.6.0.999).

### Quantification and statistical analysis

In this paper, all pre-processing analysis on the metabolomics and diet data was done in R (v4.3.0), while the machine learning and explainable AI tasks were performed in Python (v3.9.13).

Significance was determined by P-values adjusted for FDRs according to the Benjamini-Hochberg principle^140^ because it is less strict; values falling below the critical FDR of 0.05 were considered significant. Significance stars were displayed on plots, when applicable, according to the significance level (∗∗∗: FDR < 0.001, ∗∗ 0.001≤ FDR < 0.01, ∗: 0.01≤ FDR < 0.05).

In the ML tasks, the datasets were stratified by the target class when splitting between test and train, and across folds. The performances of the models in python were represented as the mean AUC score across the folds with the 95% confidence interval, while the LASSO regression task in R was represented as mean AUC score with SD and ages of the participants represented as median and SD (Table 1). Only individuals with complete diet data were included, as well as only those that already had IBD at baseline.

Differences between the IBD and non-IBD groups were calculated using either the Wilcoxon rank sum test or the Chi-square test, depending on the nature of the data (refer to Table 1; Figure S4). Although both transformed datasets had a normal distribution, confirmed by the shapiro-wilk,^141^ non-parametric tests like the Wilcoxon rank sum test and spearman-based correlation method were used due to their robustness to outliers and independence from specific distributional assumptions.

## Supplemental information

Document S1. Figures S1–S5

Table S1. UKBB plasma metabolite descriptions, related to Figures 3, 4, S1–S3, and S5

Table S2. HMP2 fecal metabolite descriptions, related to Figures 3, 4, S1–S3, and S5

Table S3. Diet data details for the UKBB and HMP2, related to Figures 4 and S4

Table S4. UKBB metabolite-diet correlation and FDR table for the IBD and non-IBD groups, related to Figure 4

Table S5. HMP2 metabolite-diet correlation and FDR table for the IBD and non-IBD groups, related to Figure 4
