# Supplementary material for: Explainable AI-prioritized plasma and fecal metabolites in inflammatory bowel disease and their dietary associations
Source: iScience. 2024 Jun 17;27(7):110298. doi: 10.1016/j.isci.2024.110298 (PMC11261406; doi:10.1016/j.isci.2024.110298)
Supplement: Document S1. Figures S1–S5 [file mmc1.pdf]

iScience, Volume *27*

Supplemental information

Explainable AI-prioritized plasma and fecal metabolites in inflammatory bowel disease and their dietary associations

Serena Onwuka, Laura Bravo-Merodio, Georgios V. Gkoutos, and Animesh Acharjee

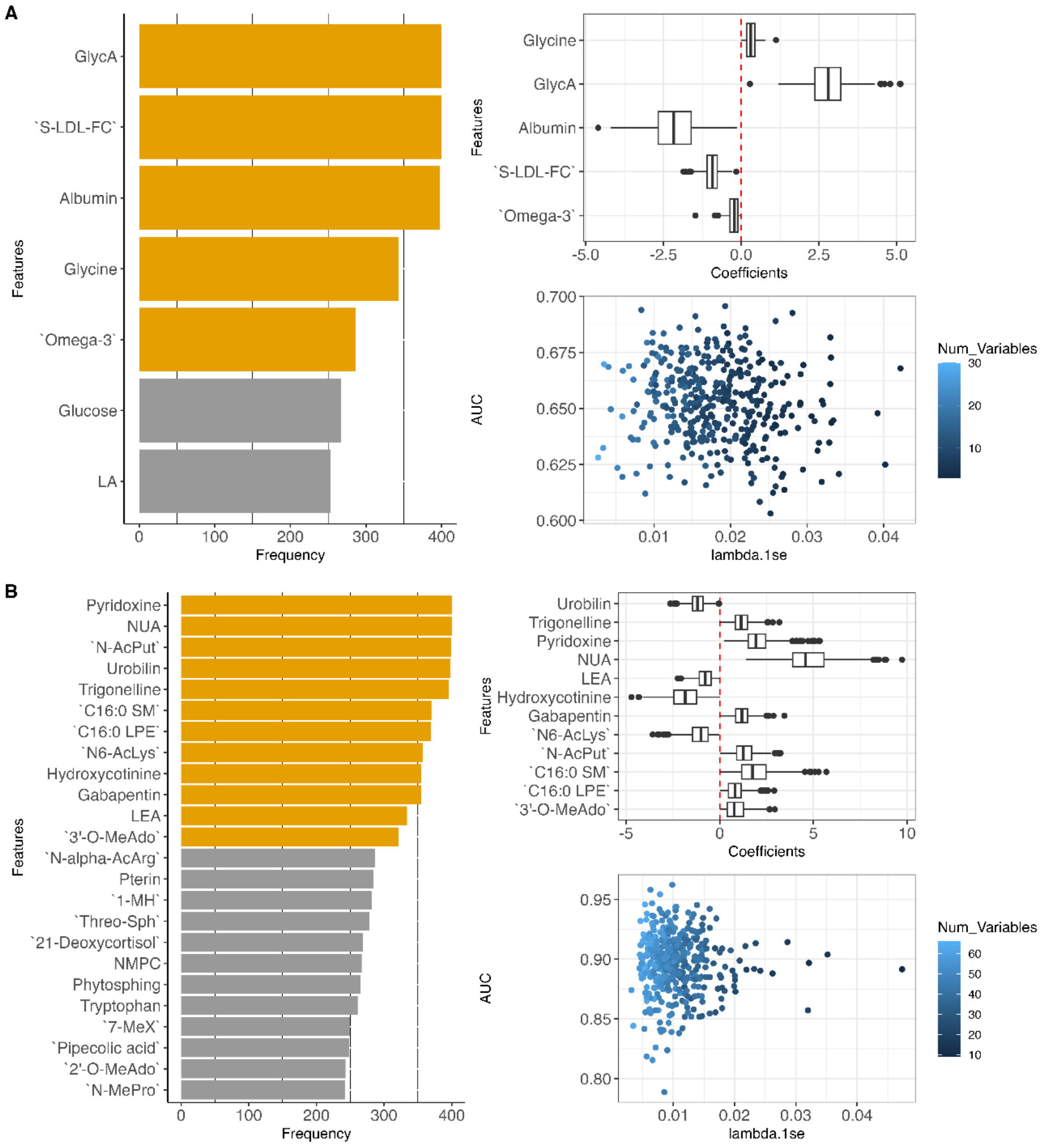

Figure S1. Results from 400 different bootstrapped LASSO models (optimized through ten-fold cross validation each) of the training split of the (A) UKBB data and the (B) HMP2 data. The left bar plot displays the ranked robustness of a feature, calculated by the number of times it appears in a model, with those features appearing more times than a threshold (average between the fourth and fifth quantile) selected. Coefficients associated to these features in all 400 models are then plotted as boxplots in upper right plot, revealing the association between IBD diagnosis and metabolite. All AUC values are found in the lower right dot plot, comparable to the AUC scores of LASSO in python. **Related to Figures 2 and 3.**

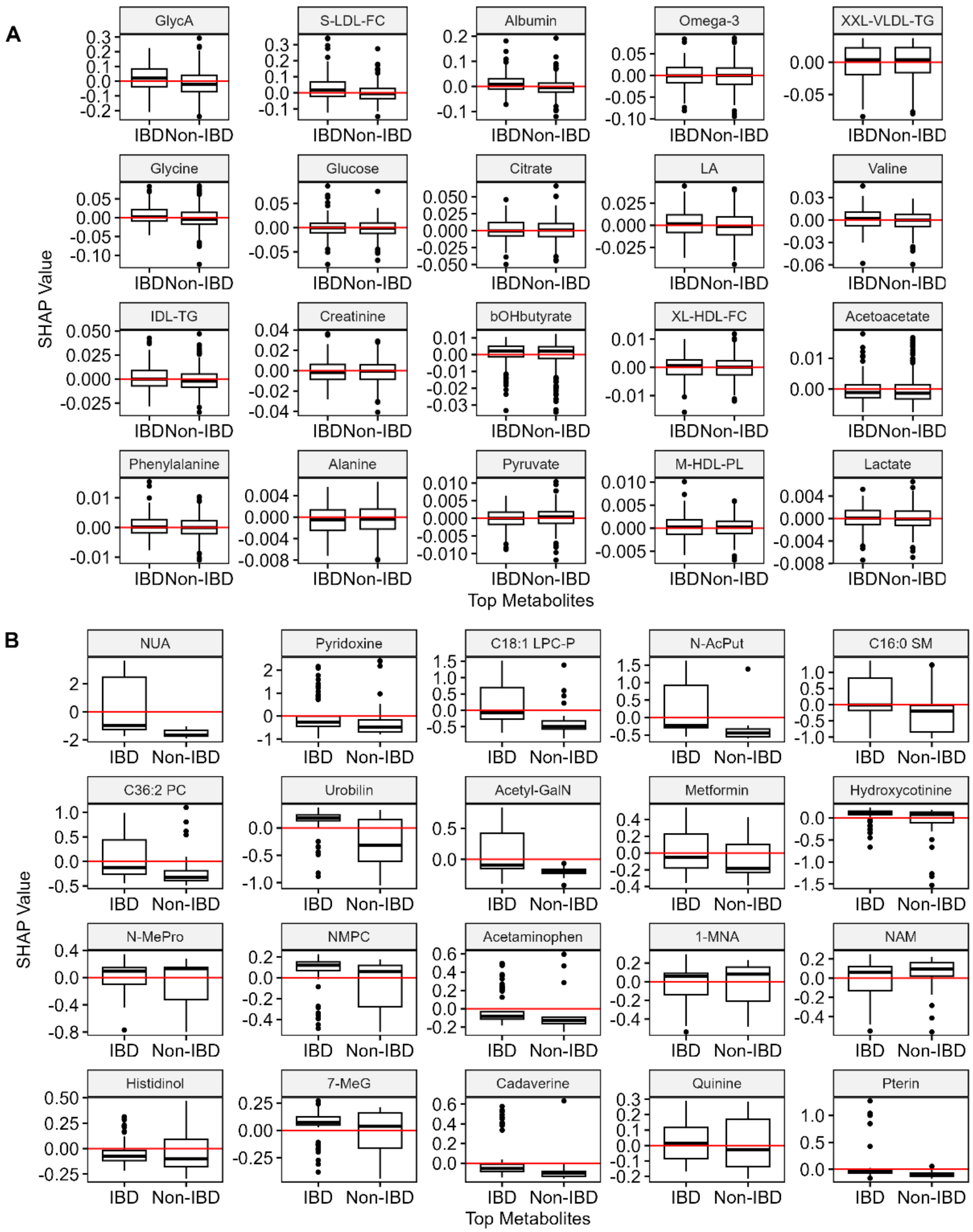

Figure S2. SHAP values of each sample in the test set of both the IBD and non-IBD classes of the (A) UKBB and (B) HMP2 cohorts for the top 20 SHAP-calculated discriminatory metabolites. Related to Figure 3. The red line represents the vertical line in the SHAP local importance summary plot in Figure 3. This line represents the point at which there is no contribution to either IBD (positive) or non-IBD (negative) prediction. A positive SHAP value for a sample implies that the sample contributed to the prediction of IBD. The magnitude of the positive value indicates the strength of this contribution. Conversely, a negative SHAP value suggests that the sample contributed to the prediction of non-IBD, with a greater negative value indicating a more substantial contribution. **Related to Figure 3**.

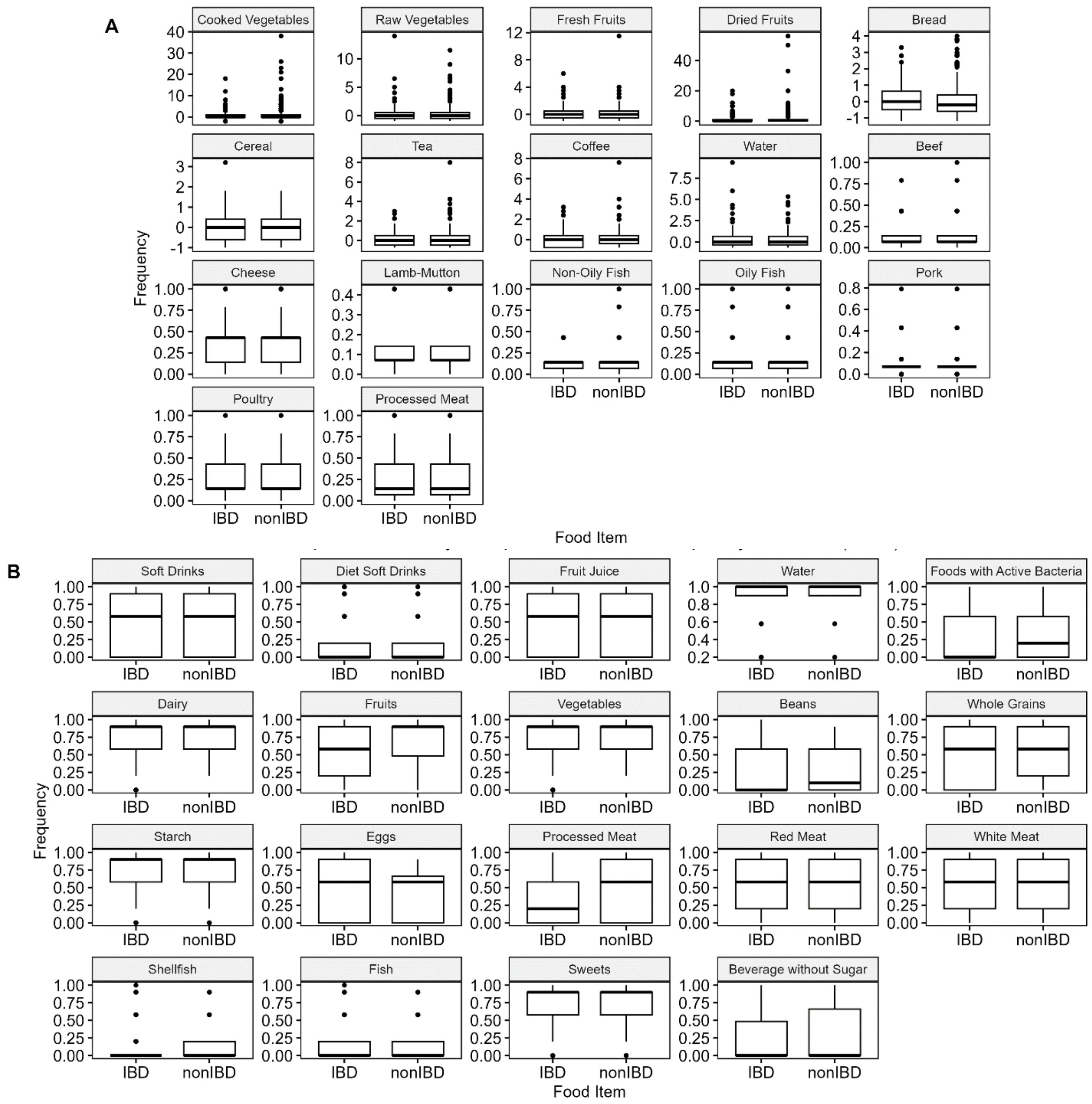

Figure S3. Food Intake Frequency boxplots in IBD versus non-IBD in the (A) UKBB and (B) HMP2 cohorts. The food items that have frequency upper limits higher than one, are the numeric dietary features, while the features with an upper frequency limit of one or less than, are the categorical incremental dietary features represented as numbers. **Related to Figure 4, Table S3, and STAR Methods.**

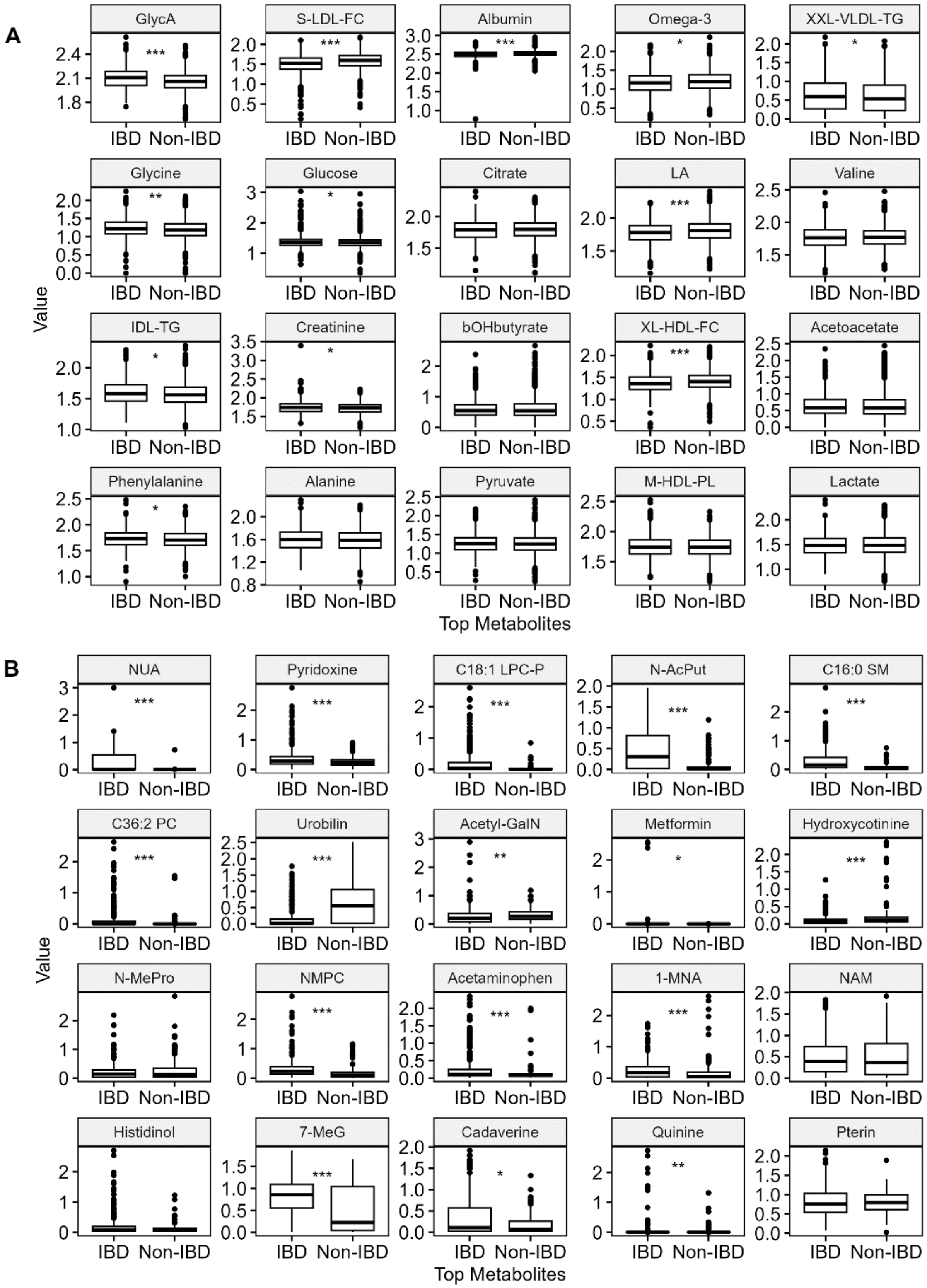

Figure S4. Plasma metabolite profile differences between IBD and non-IBD in the (A) UKBB and (B) HMP2 cohorts, for the top 20 discriminatory metabolites based on SHAP calculations. Significant differential metabolites, based on false discovery rates, are indicated with stars (***: FDR < 0.001, ** 0.001≤ FDR < 0.01, *: 0.01≤ FDR < 0.05). **Related to Figure 4.**

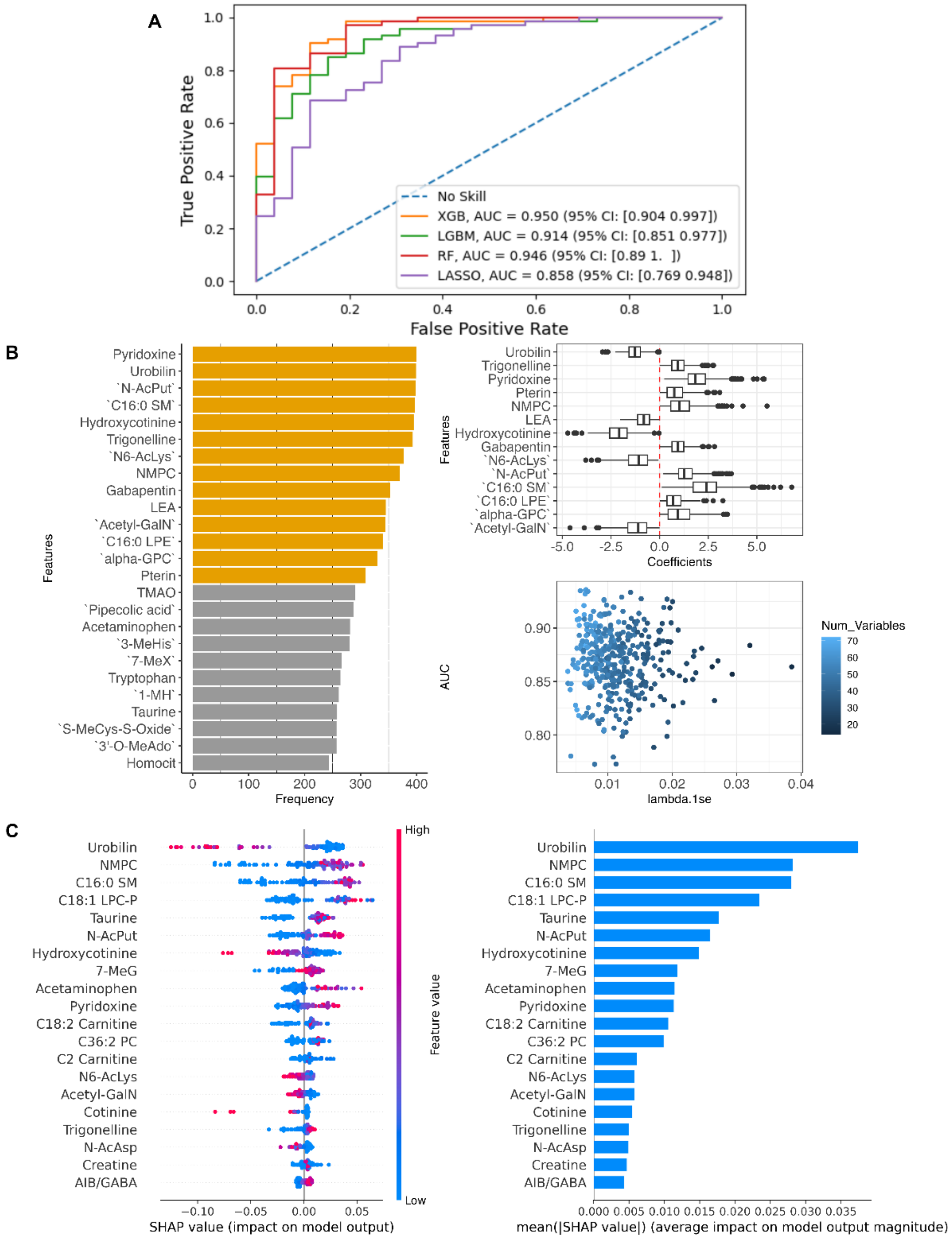

Figure S5. Illustration of the HMP2 data analysis excluding the NUA feature. (A) showcases the AUC$_{test}$-ROC curves of optimized classifiers (XGB: extreme gradient boosting also known as XGBoost, LGBM: light gradient boosting machine, RF: random forest and LASSO: least absolute shrinkage and selection operator). (B) presents results from 400 Bootstrapped LASSO models; the left bar plot indicates the frequency of feature inclusion in models, with features appearing more times than a threshold (average between the fourth and fifth quantile) highlighted; boxplots in the upper right plot display coefficients associated with these features across all 400 models; AUC values are summarized in the lower right dot plot. (C) displays SHAP summary plots of the top 20 ranked features based on global performance of the RF Model* in predicting IBD using the test set. The local importance plot, situated on the left, illustrates feature importance on a sample-level, while the global importance plot, the right bar plot, summarizes the features that are most influential on average. *RF model used instead of XGBoost due to a current issue with the SHAP explainer function and the XGBoost classifier. **Related to Figures 2 and 3.**
